# Integrating Primary and Metastatic scRNA–Seq and Bulk Data to Develop an Immune–Based Prognosis Signature for Colorectal Cancer

**DOI:** 10.3390/cimb47080652

**Published:** 2025-08-13

**Authors:** Kaiyuan Xing, Liangshuang Li, Yingnan Ma, Jiang Zhu

**Affiliations:** School of Medical Informatics, Daqing Campus, Harbin Medical University, Daqing 163319, China; xky1922@163.com (K.X.); liliangshuang1997@163.com (L.L.); mayingnan2020@outlook.com (Y.M.)

**Keywords:** colorectal cancer, scRNA–seq, metastasis, immune, prognosis, biomarkers

## Abstract

Colorectal cancer (CRC) is a highly aggressive cancer, with its treatment and prognosis particularly challenging due to metastasis. The immune response is involved in the whole process of CRC development, and immunotherapy has increasingly become a part of CRC patients’ treatment. However, comprehensive research on the immune microenvironment driving CRC metastasis remains limited. Given this limitation, we proposed a bioinformatics method to construct a metastasis–based immune prognostic model (MIPM) by integrating CRC single–cell RNA sequencing (scRNA–seq) and bulk data. Our study identified several MIPM genes significantly associated with CRC metastasis and progression. MIPM reliably predicted overall survival (OS) and tumor recurrence in CRC across eleven bulk validation datasets. Notably, MIPM could independently predict outcomes beyond traditional clinical factors such as age, sex, and stage. It showed high predictive accuracy in CRC patients treated with chemotherapy. Drug sensitivity and multifaceted immune analyses further underscored the importance of MIPM in therapeutic and immunotherapy response modulation. In conclusion, our findings have profound implications for the illustration of MIPM, which could serve as a new plausible prognostic marker for CRC patients and provide new insights for treatment strategies. The further evaluation and investigation of MIPM will enhance the prognosis and precision therapy of CRC patients.

## 1. Introduction

Colorectal cancer (CRC), the third most prevalent malignancy globally, significantly impacts global health [1,2]. Metastasis is the primary cause of CRC–related mortality, with the liver (~50% of cases) being the predominant site, followed by lymph nodes and lungs [3,4,5]. Although treatment methods like surgery and chemotherapy have improved the survival outcome of CRC patients, metastatic CRC still carries a dismal prognosis, with a 5-year overall survival (OS) rate of just 14% [6,7]. This highlights the critical need for research focused on identifying metastasis-associated genes to enhance patient prognosis and facilitate the development of personalized treatment strategies.

The tumor microenvironment plays a crucial role in tumor biology [8,9]. Immunotherapy and related combination therapies have offered hope for patients with malignant tumors, especially for CRC [10,11,12]. Therefore, many studies have recently focused on developing immune–related prognostic biomarkers for CRC patients. For example, Li et al. constructed a CRC–specific immune–related gene prognostic index (IRGPI). They demonstrated the ability of IRGPI to predict patient outcomes in the different risk groups [13]. Based on the study of the expression of immune–related and inflammatory markers in CRC, An et al. found that *APRIL*/*TNFSF13*, *BAFF*, and *MMP-3* were highly expressed in CRC, which may serve as diagnostic or prognostic markers for CRC [14]. An immune risk model was also developed by integrating immune cells and immune–related genes, which could effectively predict the prognosis of CRC patients undergoing chemotherapy [15]. These analyses have significantly advanced the field of immune–related prognostic studies.

Nevertheless, despite the promise of immunotherapy in CRC, its effectiveness remains limited by the heterogeneity of the tumor immune microenvironment (TIME), often resulting in drug resistance, recurrence, and poor prognosis. Meanwhile, while numerous factors influence the effectiveness of immunotherapy, there is lack of reliable markers to predict patient prognosis. Advancements in single–cell RNA sequencing (scRNA–seq) have greatly facilitated the identification of immune–related biomarkers across various cancers by integrating both scRNA–seq and bulk data. For instance, Zhang et al. developed a prognostic risk score based on the combination of scRNA–seq and bulk data from CRC patients. They found its strong correlation with immunotherapy sensitivity, which was conducive to developing more accurate treatment strategies for CRC patients [16]. Similarly, Liu et al. developed an immune prediction model that has significant prognostic value by integrating bulk and scRNA–seq data and they observed that it could precisely discriminate the immune status of patients [17]. Wang et al. integrated the scRNA–seq data with bulk data to construct a prognostic model, whose prognostic efficacy was validated in five independent cohorts [18]. However, the absence of robust immune and metastasis prognostic markers remains a major challenge in oncology. Constructing prognostic signatures related to immunity and metastasis is of paramount importance in the fight against cancer and other metastatic diseases. These signatures enable clinicians to predict disease progression more accurately and develop new immunotherapeutic strategies, ultimately leading to improved survival rates and better outcomes for patients.

In the study, we utilized CRC bulk and scRNA–seq data, including primary and matched metastases samples to develop a metastasis–based immune prognostic model (MIPM) that effectively predicts CRC prognosis. This MIPM’s robust predictive capabilities for OS, tumor recurrence, and chemotherapy benefits in CRC patients were validated in eleven datasets. Additionally, the MIPM’s associations with clinical characteristics and drug sensitivities were explored, and we also evaluated the immune checkpoint inhibitors (ICIs) therapy response in MIPM subgroups, which together established its potential as a significant prognostic biomarker. Our findings highlight MIPM as a crucial tool for advancing CRC metastasis and immune research, potentially guiding more precise treatment approaches for CRC patients.

## 2. Materials and Methods

### 2.1. CRC scRNA–Seq and Bulk Data Acquisition and Pre–Procession

Colorectal cancer (CRC) single–cell RNA sequencing (scRNA–seq) data from GSE178318, encompassing 113,331 cells, were downloaded from the Gene Expression Omnibus (GEO) database (https://www.ncbi.nlm.nih.gov/geo/ (accessed on 1 June 2022)), which contains primary and matched liver metastasis samples derived from six CRC patients (Table 1) [19,20]. Three of the six patients (specifically those identified as COL15, COL17, and COL18), received preoperative chemotherapy, while the others were treatment–naïve. To capture a broad range of cellular responses and gene expressions relevant to both treatment–naïve and treated CRC conditions, we aggregated and analyzed the scRNA–seq data from all six patients. The raw expression data with unique molecular identifier (UMI) counts were normalized and scaled using the R package “Seurat” (version 5.0.1), while low–quality cells and genes were filtered out as follows: (1) the exclusion of cells with <300 expressed genes or >15% mitochondrial gene content; (2) filtered out mitochondrial genes and ribosomal genes, and genes expressed in <3 cells; (3) retention of protein–coding genes. We stratified cell clusters and annotated them based on the known marker genes from Che et al. (Supplementary Table S1). According to the annotation results, the expression data of cancer cells were extracted for downstream processing.

The clinical and expression data of 1836 patients of 11 CRC bulk datasets were obtained from the GEO database, including GSE39582 [21], GSE159216 [22], GSE87211 [23], GSE29621 [24], GSE72968 [25], GSE72970 [25,26], GSE12945 [27], GSE17536 [28], GSE17537 [28], GSE17538 [28], GSE37892 [29] (Table 1). All selected bulk expression datasets were log2-transformed.

### 2.2. Identification of Gene Expression Signature from Primary and Metastatic Cells

To further distinguish primary and metastatic cancer cells, the signal–to–noise statistics for identifying feature genes that could be used to classify these two group cells were calculated. The signal–to–noise statistic for each gene was calculated by

(1) $$S_{i}=\left(\mu_{p}-\mu_{m}/\sigma_{p}+\sigma_{m}\right)$$

where $S_{i}$ represents the signal–to–noise statistic for gene i, $\mu_{p}$ and $\mu_{m}$ separately represent the mean expression values of gene i in primary and metastatic cells, and $\sigma_{p}$ and $\sigma_{m}\;$represent the standard deviation of gene i in primary and metastatic cells.

Genes were ranked by the signal–to–noise statistic, followed by weighted–voting classification and ‘leave–one–out’ cross–validation algorithm to determine the optimal gene set distinguishing primary and metastatic cells [30,31]. Specifically, given a dataset with N cells, each time leaving out one cell as the test set and using the remaining N−1 cells for training. First, calculate the $S_{i}$ of each gene in the signature in the training set. Next, the weighted–voting classification algorithm found the decision boundaries between the primary and metastatic means for each gene:

(2) $$b_{i}\;=\;(\mu_{p}-\mu_{m})/2$$

For a test sample x in cross–validation analysis, each gene i in the feature gene set casts a vote:

(3) $$v_{i}\;=\;S_{i}(g_{i}^{x}-b_{i})$$

where $g_{i}^{x}$ represents the expression value of gene i in sample x. And, $\sum v_{i}$ is used for the final vote for primary or metastases.

Finally, the feature gene set with the highest cross–validation accuracy was selected as the most discriminating gene expression signature.

### 2.3. Screening High–Contribution Immune–Related Genes

A total of 2013 immune–related genes were downloaded from the ImmPort (https://www.immport.org/resources (accessed on 8 May 2024)) database [32]. CRC patients in GSE39582 were divided into high–immunity and low–immunity groups based on the median immune score which was calculated by a single sample gene set enrichment analysis (ssGSEA) method. Then, we used the random forest method to calculate the contribution of 2013 immune genes, and extracted genes with mean decrease Gini > 1 (refer to the analysis of Shen et al.) as high–contribution immune–related genes (HIRGs) [33].

### 2.4. Construction of Metastasis–Based Immune Prognostic Model (MIPM)

We intersected the most discriminating gene expression signature and HIRGs. Subsequently, in the GSE39582 dataset, we used univariate Cox regression analysis to identify prognosis–related genes (*p* < 0.05) significantly correlated with the survival among the intersected genes. These identified prognosis–related genes were used for the subsequent construction of prognostic model. Lasso regression is a type of penalized regression technique commonly used for variable selection and dimensionality reduction. It is based on the minimum mean cross–validated error criterion and determines the optimal value of the tuning parameter (λ) through ten–fold cross–validation. Then, based on the expression profiles of each sample, Lasso selected the prognosis–related genes that met the criteria and assigned a corresponding coefficient to each, which is then used to establish MIPM:

(4) $$MIPM\;risk\;score=\sum_{i=1}^{n}r_{i}Exp\left(i\right)$$

where $r_{i}$ is the regression coefficient of gene i, n is the number of MIPM genes, and $Exp\left(i\right)$ is the expression value of gene i in sample.

### 2.5. Functional Enrichment Analysis

Based on the canonical pathway gene set collections of the MSigDB database (https://www.gsea-msigdb.org/gsea/msigdb/ (accessed on 14 May 2024)), we conducted the enrichment analysis for intersection genes and MIPM genes utilizing the “clusterProfiler” package (version 4.10.0) [34].

### 2.6. Evaluation of MIPM in Validation Cohorts

The prognostic power of MIPM was evaluated in 11 validation cohorts. The cutoff points were defined by the “survminer” package (version 0.4.9) and the differences in overall survival (OS) for patients were compared using Kaplan–Meier (K–M) curves with the log–rank test [35]. Subsequently, the decision curve analysis (DCA), calibration curves, and receiver operating characteristic (ROC) curves were used to evaluate the predictive ability and application value of MIPM. Univariate and multivariate Cox regression analyses were used to assess independent prognostic factors. The comprehensive nomograms were built to predict the survival of CRC patients, and the calibration curves and ROC curves to validate the accuracy of the nomograms for predicting the OS of CRC patients at 3– and 5–year follow–up [36,37]. Additionally, we evaluated the ability of MIPM to predict chemotherapy benefits and tumor recurrence outcomes (disease–free survival, DFS) in validation datasets by prognostic analysis. We further compared the prognostic performance of MIPM with several previously published immune–related prognostic models (including IRGPI and models proposed by Xiao et al., Zheng et al., and Li et al.) [13,38,39,40]. In all 11 validation datasets, we calculated a risk score for each sample based on the respective model (with detailed formulas provided in Supplementary Table S2), and then compared the OS differences between risk groups and AUC values of the predictive efficacy.

### 2.7. Analysis of Clinical Features

In the GSE39582 dataset, we portrayed the differences in MIPM genes expression and clinical features (including age, sex, stage, chemotherapy, survival time, and survival event) between high– and low–risk groups. The ROC curves were applied to compare the accuracy of MIPM to other clinical variables (age, sex, stage) in predicting patients’ survival. Additionally, we also assessed differences in MIPM risk scores between different clinical variables and consensus molecular subtypes (CMSs), and the prognostic efficacy of MIPM in CMS subtypes.

### 2.8. Drug Sensitivity Analysis

Expression and drug sensitivity data (the half maximal inhibitory concentration (IC50)) for 44 CRC cell lines were downloaded from the Genomics of Drug Sensitivity in Cancer (GDSC) (https://www.cancerrxgene.org/ (accessed on 1 June 2024)) database [41]. We used the oncoPredict method to predict the sensitivities of patients in the high– and low–risk groups in the GSE39582 dataset to drugs [42]. We linked the predicted drug sensitivities to enriched pathways to support the prediction conclusion. Spearman correlation analysis was applied to further examine the associations between MIPM risk scores and drug sensitivity to 286 compounds.

### 2.9. Comprehensive Immune-Related Analysis

Using CIBERSORT, we quantified 22 immune cell signatures in GSE39582 and compared their infiltration levels between high– and low–risk groups [43,44]. We utilized the tumor immune dysfunction and exclusion (TIDE) method to assess the immunotherapy response, and described the differences in TIDE scores among different MIPM subgroups [45]. We further assessed the correlations between risk scores and immune–related characteristics. Finally, we employed the immunophenoscore (IPS) to quantify patient response to immune checkpoint therapy (PD1 and PD–L1), comparing the differences of IPS between risk groups [46,47].

### 2.10. Statistical Analysis

All the statistical analyses were performed in R software (version 4.3.1). The Wilcoxon test was used to compare the differences in values between the test and control groups. A difference of *p* < 0.05 was regarded as statistically significant.

## 3. Results

### 3.1. Single–Cell RNA Sequencing (scRNA–Seq) Data Acquisition and Processing

**Figure 1:**
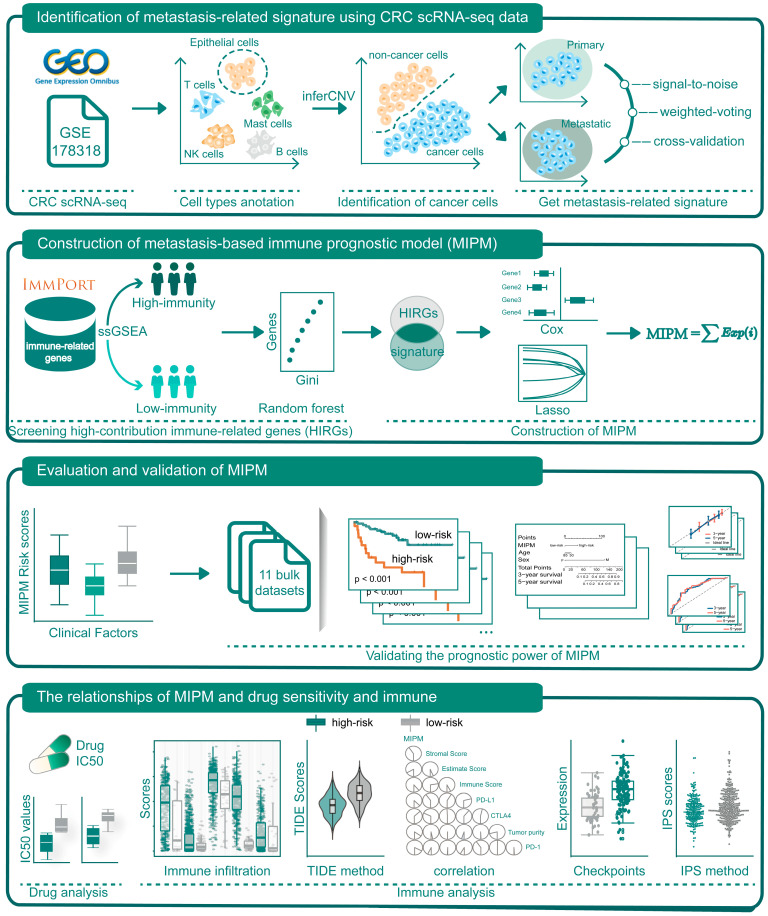
The workflow of this study.

**Figure 2:**
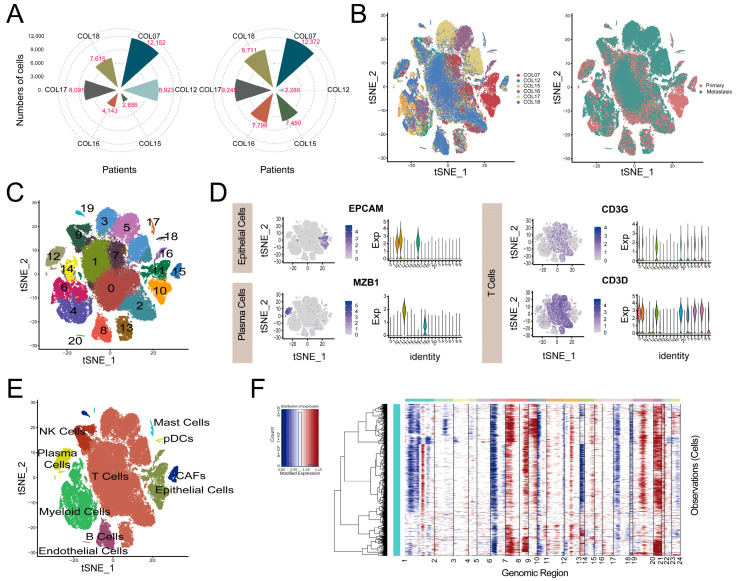
Single–cell atlas of CRC. (**A**) Patient origin of cells in primary (left) and liver metastases (right) tumors. (**B**) T–SNE plot for cells in distinct patients (left) or sites (right). (**C**) Clustering of single cells and label colors according to separate clusters. (**D**) The plots of typical markers of some main cell types (epithelial/plasma/T cells). (**E**) Identification of main cell types based on the expression of marker genes, label colors by cell types. (**F**) Heatmap of large–scale CNVs to distinguish cancer cells from non–cancer cells.

The largest scRNA–seq dataset of GSE178318 was downloaded for the construction of the metastasis–based immune prognostic model (MIPM) for the criterion containing both primary and matched liver metastasis samples. The flowchart of this study is shown in Figure 1. After quality control (QC), as described in the Methods section, a total of 92,675 single cells (48,865 cells in the liver metastases and 43,810 cells in the primary tumor) from different patients with 16,499 protein-coding genes were obtained, which were shown in Figure 2A. T–distributed stochastic neighbor embedding (t–SNE) was used for nonlinear dimension reduction, as shown in Figure 2B. Then, these single cells were stratified into 21 clusters, and we used marker genes to annotate the main cell populations, including T cells, epithelial cells, plasma cells, myeloid cells, CAFs, mast cells, pDCs, endothelial cells, B cells, and NK cells (Figure 2C–E). Next, the inferCNV method was utilized to identify 7587 cancer cells from all epithelial cells, and the expression profiles of these cancer cells were extracted for further analysis (Figure 2F).

### 3.2. Establishment of Metastasis–Based Immune Prognostic Model (MIPM)

**Figure 3:**
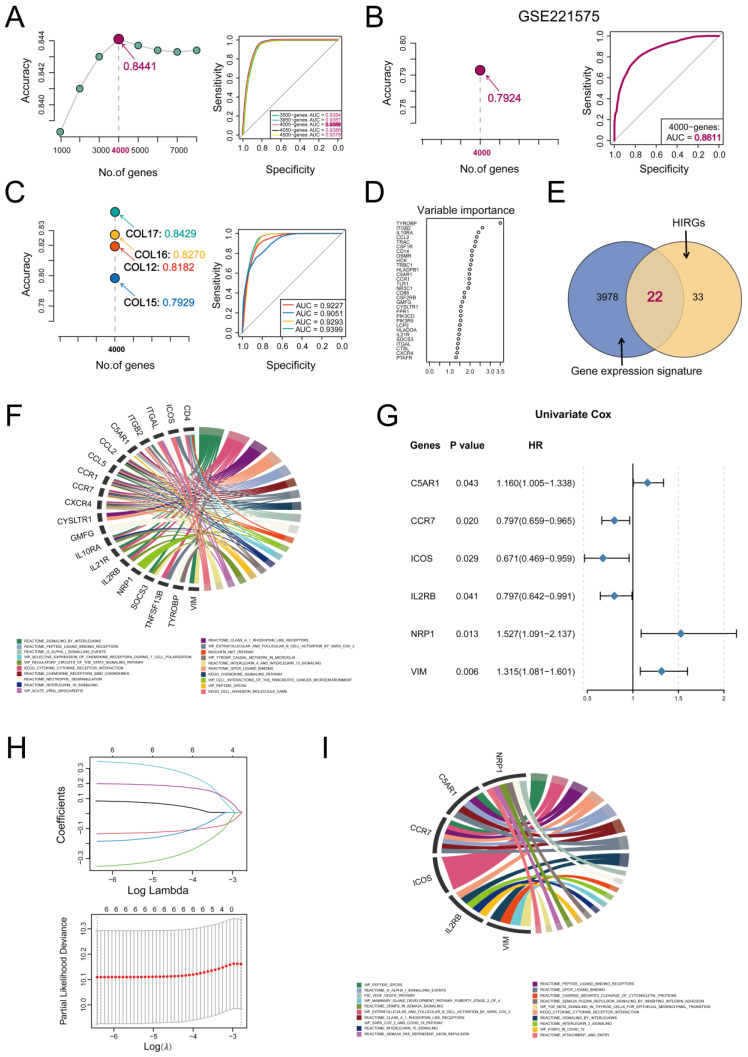
Development of metastasis–based immune prognostic model (MIPM). (**A**) Plot for the prediction accuracy of multiple gene signatures distinguishing primary and metastatic CRC cancer cells. (**B**) To verify the classification efficacy of the 4000–gene signature in an additional scRNA–seq dataset. (**C**) Cross–validation at the patient level was used to assess the performance of the 4000–gene signature. (**D**) Immune-related genes importance plot. (**E**) Twenty–two intersection genes between metastases–associated gene expression signature and high–contribution immune–related genes. (**F**) The enrichment analysis results of twenty–two intersection genes. (**G**) Forest plot of univariable Cox regression analysis with six prognosis–related genes. (**H**) Plots of selection of MIPM genes based on Lasso regression, each curve represents the coefficient variation trajectory of each MIPM gene. (**I**) The functional enrichment analysis results of MIPM genes.

By measuring the classified ability of feature gene sets in the scRNA–seq dataset, we found a gene expression signature containing 4000 genes that had the maximum prediction accuracy (84.41%) for distinguishing primary cells from metastatic ones, and the area under the ROC curve (AUC) for evaluating the predictive accuracy of the 4000 genes reached the maximum value of 0.9388, outperforming other gene sets (Figure 3A). To further validate the robustness of the signature’s classification performance, we applied the signature to an independent scRNA–seq dataset (GSE221575). The signature consistently discriminated between primary and metastatic cells (AUC = 0.8611, accuracy = 0.7924), highlighting its stability and cross-dataset applicability (Figure 3B). In addition, we also performed cross–validation at the patient level to avoid patient–specific bias. Specifically, we used the cells from one patient as the test set and the cells from the remaining patients as the training set to predict the types of cells from that held–out patient. The results consistently demonstrated the strong classification power of the signature. For example, when using patient COL12/COL15 as the test set, it achieved an accuracy of 0.8182/0.7929 and an AUC value of 0.9227/0.9051 (Figure 3C). Given the significant relationship between the immune landscape of cancer and the prognosis of the patient, we employed enrichment scores derived from 2013 immune–related genes to stratify CRC patients into high–immunity and low–immunity groups, and selected 55 high–contribution immune–related genes (HIRGs) with a mean decrease in Gini > 1 by the random forest method (Figure 3D). Subsequently, 22 intersection genes between metastases–associated gene signature and HIRGs were obtained (Figure 3E). Functional enrichment analysis revealed that these intersection genes were mainly enriched in canonical pathways such as “signaling by interleukins”, “cytokine–cytokine receptor interaction”, “chemokine receptors bind chemokines”, “cell interactions of the pancreatic cancer microenvironment”, and “selective expression of chemokine receptors during T cell polarization” (Figure 3F).

Subsequently, we used univariate the Cox regression analysis method to identify six genes that were significantly correlated with CRC patients’ overall survival (OS) from intersection genes (Figure 3G,H), and by Lasso regression analysis, six genes were selected to establish MIPM using the expression of genes weighted by the Lasso regression coefficient as follows: MIPM risk score = (0.077 × exp(*C5AR1*)) + (−0.141 × exp(*CCR7*)) + (−0.359 × exp(*ICOS*)) + (−0.191 × exp(*IL2RB*)) + (0.340 × exp(*NRP1*)) + (0.191 × exp(*VIM*)).

The results of the functional enrichment analysis of MIPM genes indicated that MIPM was mainly involved in the pathways “cytokine–cytokine receptor interaction”, “GPCR ligand binding”, “signaling by Interleukins”, “caspase–mediated cleavage of cytoskeletal proteins”, etc. (Figure 3I).

Notably, we found that six MIPM genes were potentially involved in immune regulation, metastasis, and the progression of CRC. Among them, *C5AR1* was found to be able to predict poor survival in CRC patients, and it played a prominent role in tumorigenesis and the development of CRC by modulating the immune response [48]. A previous study using an animal model of CRC demonstrated that *CCR7* expression was associated with lymph node metastasis [49]. Zhang et al. found a significant negative correlation between *ICOS* expression and enhanced survival of CRC patients, especially in the case of tumor metastasis, suggesting that *ICOS* may be a useful predictor of progression in CRC patients [50]. Additionally, *IL2RB* was discovered as the most common gene associated with immune checkpoint genes in CRC, and the potential predictive value of *IL2RB* for immune checkpoint therapy response was investigated [51]. A much higher expression level of *NRP1* was discovered in metastatic CRC tumors than in primary, and the knockdown of *NRP1* also has strong inhibitory effects on the metastasis of CRC cells [52,53]. *VIM* was known as a potential cancer therapeutic target, and reports found that *VIM* could promote the invasion and metastasis of CRC cells by binding to overexpressed *FSTL1* [54]. These findings further demonstrated that the MIPM was closely related to CRC.

### 3.3. Associations of MIPM and Clinical Characteristics in Internal Validation Datasets

**Figure 4:**
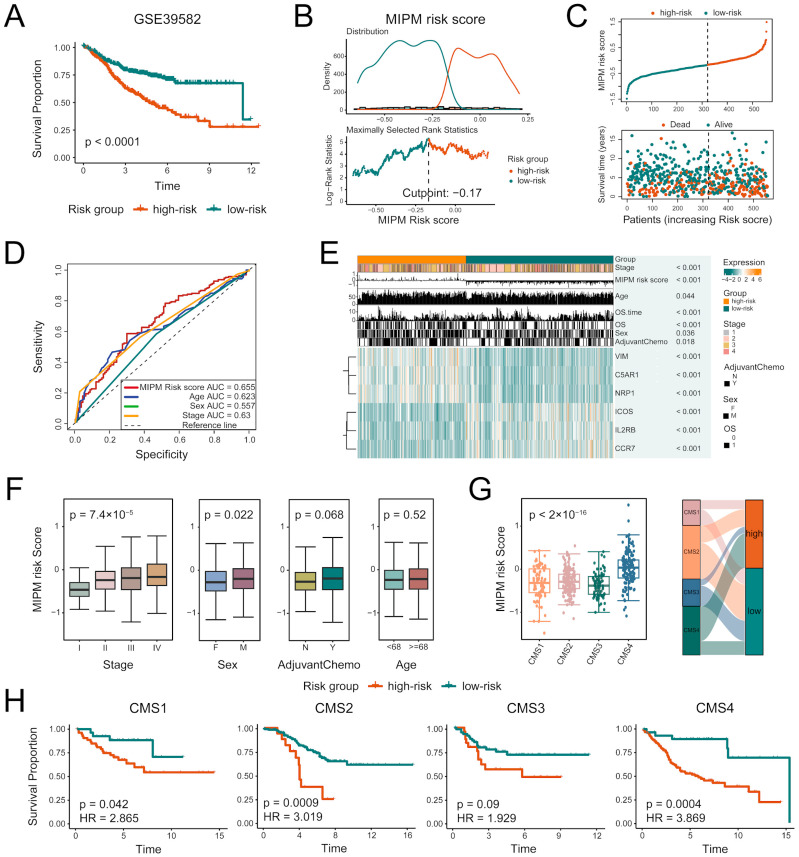
Associations between MIPM and clinical characteristics. (**A**) Kaplan–Meier survival curves of OS between high–risk and low–risk patients in GSE39582 datasets were used to compare the survival differences between the two groups. (**B**) The optimal threshold for grouping determined by the “survminer” method is −0.17. (**C**) Risk curve and risk scatter plot of CRC patients demonstrated a clear separation between high–risk and low–risk patients. (**D**) ROC curves of MIPM risk scores and other clinical variables (age, sex, stage) for predicting OS. Among them, MIPM had a better predictive ability compared with other clinical variables. (**E**) The heatmap showed significant differences in MIPM gene expression and various clinical features (age, sex, stage, etc.) between high− and low−risk groups. (**F**) The MIPM risk scores exhibited significant differences among subgroups with different clinical features. (**G**) There were differences in the MIPM risk scores among CMS groups, and Sankey map further demonstrated the corresponding relationships between CMS groups and MIPM risk groups. (**H**) The prognostic stratification ability of MIPM for patients with CMS subtypes in GSE39582 datasets.

When the MIPM was applied to the internal validation dataset (GSE39582) to assess the prognostic value of MIPM in terms of OS, 556 patients were divided into high–risk and low–risk groups using the cutoff value (−0.17), and patients in the high–risk group had significantly shorter OS time than those in the low–risk group (*p* < 0.0001) (Figure 4A–C). The results of the receiver operating characteristic (ROC) curves showed that MIPM had the highest accuracy in predicting patients’ survival compared to other clinical variables (age, sex, stage) (Figure 4D). There were significant differences in MIPM genes expression and various clinical features between high– and low–risk groups (*p* < 0.05) (Figure 4E). Different MIPM risk score distributions were found in stage and sex subgroups, but the differences were not significant in age and chemotherapy subgroups (Figure 4F). Previous studies have indicated that, among four consensus molecular subtypes (CMS,) for CRC, the CMS4 subtype has the worst OS [55]. So, we evaluated the MIPM risk scores in the context of CMS status, we found that high–risk patients accounted for the highest proportion in the CMS4 subtype and the MIPM risk scores were significantly higher in the CMS4 subtype than in other subtypes (*p* < 2 × 10^−16^) (Figure 4G). To evaluate whether MIPM provided additional prognostic value beyond known CMS subtypes, we performed CMS–stratified survival analyses. The MIPM exhibited significant prognostic capacity in most subtypes, indicating its robustness independent of CMS classification (Figure 4H). These results illustrated that MIPM might play an essential role in the prognostic prediction of CRC patients and there were strong relationships between it and clinical characteristics.

### 3.4. MIPM’s Prognostic Power Across Multiple External Validation Datasets

**Figure 5:**
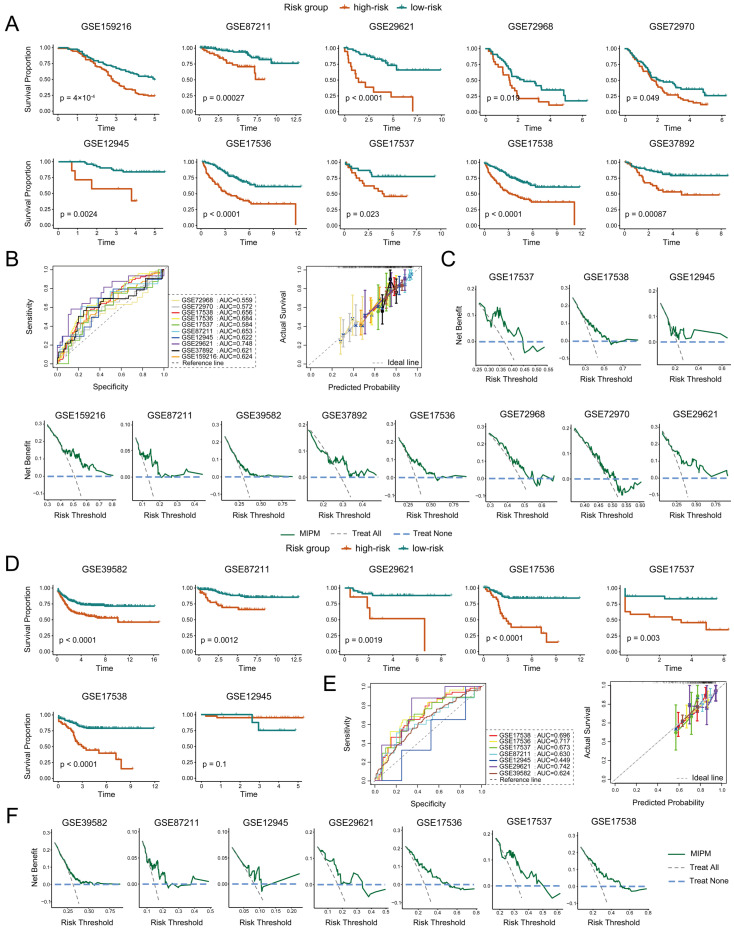
Evaluation of the prognostic efficacy of MIPM in multiple bulk validation datasets. (**A**) Kaplan–Meier survival curves were plotted in ten external validation datasets to compare the differences in OS between high–risk and low–risk patients. (**B**) ROC curves and calibration curves were used to evaluate the predictive ability (OS) of MIPM in the validation datasets. (**C**) DCA was employed to evaluate the clinical utility of the MIPM. (**D**) Assessment of the MIPM’s effectiveness in predicting recurrence risk across seven validation datasets with available tumor recurrence outcomes. (**E**) The discriminative capacity and predictive accuracy of the MIPM for DFS were validated using ROC and calibration curves, while (**F**) DCA was applied to determine its net clinical benefit.

To evaluate the robustness of MIPM, we tested its prognostic power using ten external validation datasets, including GSE159216, GSE87211, GSE29621, GSE72968, GSE72970, GSE12945, GSE17536, GSE17537, GSE17538, and GSE37892. We found that MIPM could effectively categorize patients into high− and low−risk groups with significantly different OS in all datasets, and patients with the high−risk group had worse OS outcomes (Figure 5A and Table 2). The MIPM demonstrated good and consistent prognostic performance across the validation cohorts, as indicated by AUC values in ROC analysis, great concordance between the predicted and observed outcomes in calibration curves, and substantial net clinical benefit in the decision curve analysis (DCA), highlighting its potential clinical applicability in prognostic risk stratification (Figure 5B,C). Subsequently, we also confirmed the effectiveness of the MIPM for recurrence risk prediction in seven validation datasets containing information on tumor recurrence outcomes (DFS) (Figure 5D). Similarly, MIPM maintained consistent predictive accuracy to DFS and a practical clinical benefit across validation datasets, supported by AUC, calibration, and DCA analyses (Figure 5E,F). Taken together, these findings suggested that MIPM had important clinical implications and might serve as an important biomarker for predicting survival in CRC patients.

### 3.5. MIPM Predicts Chemotherapy Benefits

**Figure 6:**
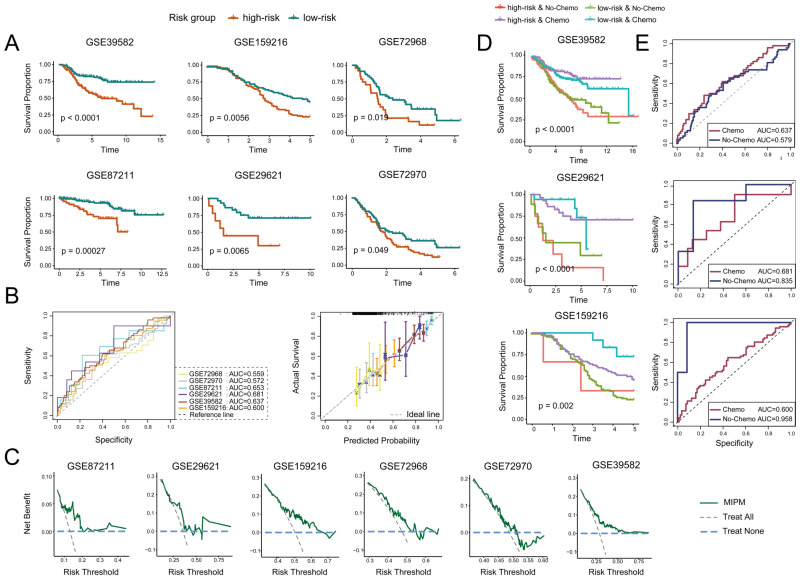
(**A**) Evaluation of the predictive power of MIPM in CRC patients receiving chemotherapy in six validation datasets containing chemotherapy information. Evaluating the ability of MIPM to predict chemotherapy benefit and its clinical applicability by (**B**) ROC, calibration curves, and (**C**) DCA. (**D**) Patients were categorized into four subgroups according to MIPM (high–risk/low–risk) and chemotherapy status (yes/no), allowing the assessment of interaction effects. (**E**) ROC curves were conducted to compare the performance of MIPM in predicting OS between chemotherapy-treated and untreated patients.

Chemotherapy significantly prolonged the OS time in CRC patients and remains a primary therapeutic strategy [56]. Therefore, we evaluated the predictive power of MIPM for CRC patients with chemotherapy in six validation datasets (Table 1). The results showed that MIPM successfully classified CRC patients receiving chemotherapy into high– and low–risk groups with markedly different OS, demonstrating that MIPM had good ability to predict OS in CRC patients who received chemotherapy (Figure 6A). In addition, ROC analysis, calibration curves, and DCA provided evidence supporting the predictive accuracy of MIPM for chemotherapy benefit and its potential value in clinical practice (Figure 6B,C). We further confirmed this finding through response stratification analyses. Chemotherapy significantly improved patients’ survival, and those who received chemotherapy exhibited a higher survival rate, indicating a substantial clinical benefit from chemotherapy (Figure 6D). Furthermore, the ROC curves revealed that MIPM’s prognostic performance remains stable regardless of chemotherapy, suggesting that MIPM can reliably predict patient risk inde-pendent of treatment status Figure 6E).

### 3.6. Independent Prognostic Factor Evaluation and Nomogram Construction

To investigate the independence of MIPM from other clinical factors, such as age, sex, and stage, univariate and multivariate Cox regression analyses were performed in three validation cohorts (GSE39582, GSE17536, and GSE17538) that included the aforementioned clinical variables (Table 3). The results from the GSE39582 cohort showed that MIPM, age, and stage were independent prognostic factors for CRC patients (*p* < 0.001). In GSE17536, MIPM and stage were associated with the OS of CRC patients (*p* < 0.001). Likewise, MIPM and stage were two independent prognostic factors for CRC patients in GSE17538 (*p* < 0.001). These results confirmed a strong association between MIPM and OS in CRC patients.

**Figure 7:**
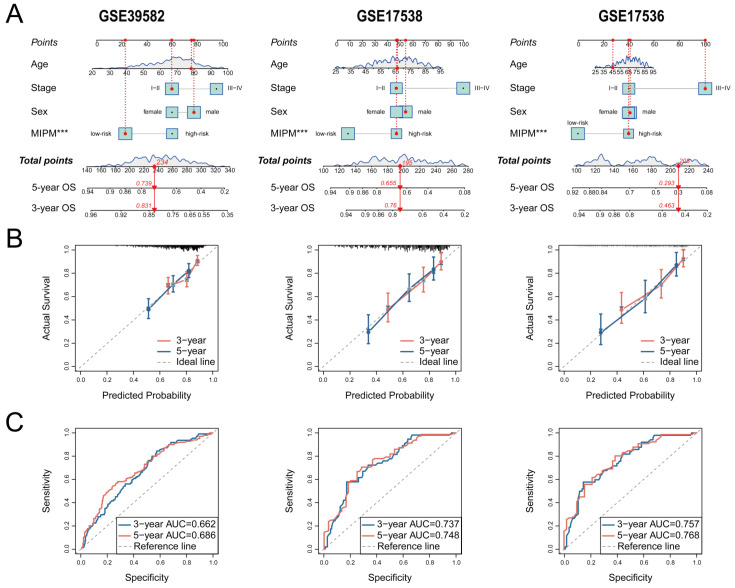
Construction and validation of nomogram models. (**A**) MIPM combined clinical variables (age, sex, stage) to build nomogram models to predict the 3– and 5–year OS of CRC patients (‘***’, *p* < 0.001). Evaluation of the predictive powers of nomogram models by (**B**) calibration curves. (**C**) ROC curves were used to test the nomograms’ predictive performance, and the results demonstrated that they achieved relatively high predictive accuracy.

Then, we designed a comprehensive nomogram based on the above variables to predict the 3– and 5–year OS of CRC patients (Figure 7A). The calibration and ROC curves were used to evaluate the predictive accuracy of the nomogram. The calibration curves displayed good agreement between predicted and actual OS, and in GSE39582, GSE17538, and GSE17536 datasets, the AUC values of 3– and 5–year were 0.662 and 0.686, 0.737 and 0.748, and 0.757 and 0.768, respectively (Figure 7B,C), which indicated that nomogram models had a good performance for OS prediction.

### 3.7. MIPM’s Influence on Drug Sensitivity and Resistance in CRC

**Figure 8:**
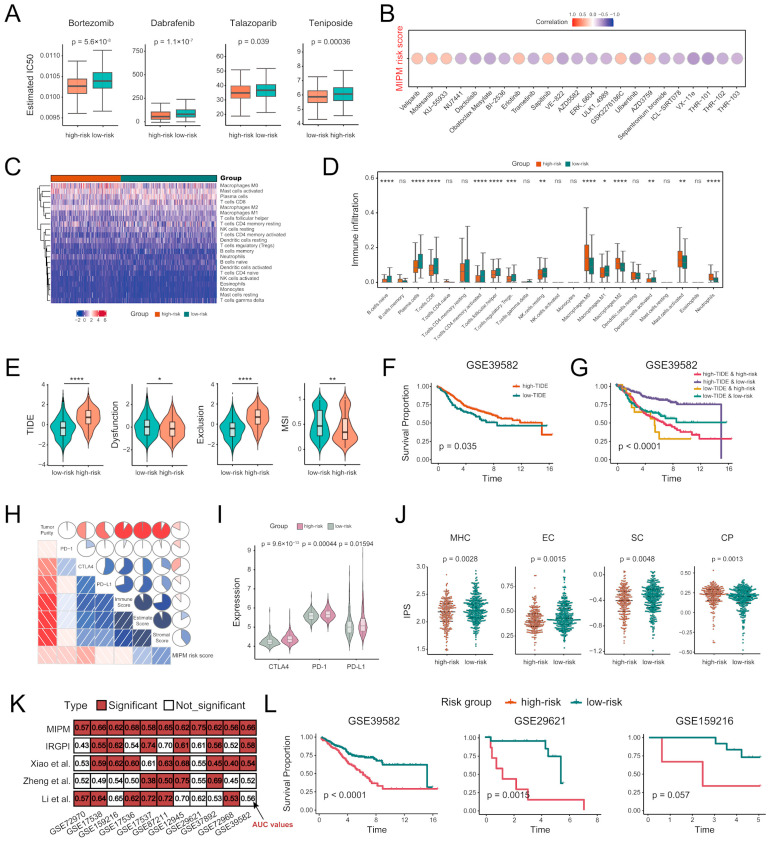
Analysis of drug sensitivity and immunity. (**A**) The predicted drug sensitivities (IC50 values) of the four drug components (Bortezomib, Dabrafenib, Talazoparib, and Teniposide) differed in CRC cell lines between high– and low–risk groups (*p* < 0.05). (**B**) The significant correlations (*p* < 0.05) between MIPM risk scores and drug sensitivities. Red (or purple) means that the cell lines with higher risk scores were resistant (or sensitive) to the drug, which suggested that these drugs might serve as potential candidates for the CRC treatment. (**C**) The abundance of signatures was calculated for twenty-two immune cell subpopulations for high– and low–risk patients. (**D**) Differences in the abundance of immune infiltration of twenty-two immune cells between high– and low–risk groups. (**E**) Comparison of the differences in TIDE, T cell exclusion, T cell dysfunction, and MSI scores in different MIPM subgroups. Differences of the OS among patient groups stratified by (**F**) TIDE scores and (**G**) MIPM risk scores and the combined TIDE scores. (**H**) The correlations between MIPM risk scores and immune–related features (stromal scores, immune scores, estimate scores, and tumor purity calculated by ESTIMATE) and the expression of key immune checkpoints (PD–1, PD–L1, and CTLA4), red represents positive correlation and blue represents negative correlation. (**I**) The expression differences of the immune checkpoint genes PD–L1, PD–1, and CTLA4 in different MIPM subgroups. (**J**) Comparison of MHC, EC, SC, and CP scores differences between different MIPM subgroups. (**K**) Comparison of the prognostic efficacy of MIPM with other immune–related prognostic models [13,38,39,40] (*p* < 0.05 is represented by ‘significant’, and the values in the figure represent the AUC values). (**L**) The predictive performance of MIPM in chemotherapy–naïve patients. Intergroup comparisons were performed using the Wilcoxon test, with the *p*–value threshold of 0.05 for statistical significance. Group allocation was explicitly annotated in the figure. ‘****’, *p* < 0.0001; ‘***’, *p* < 0.001; ‘**’, *p* < 0.01; ‘*’, *p* < 0.05; ‘ns’, 0.05 < *p*.

To enhance the clinical utility of MIPM, we performed drug sensitivity prediction and correlation analysis. The results of drug sensitivity analysis predicted by the oncoPredict method in the GSE39582 datasets showed that significantly higher drug sensitivity in high–risk patients to several drugs, including Bortezomib, Dabrafenib, Talazoparib, and Teniposide (Figure 8A). To explore the mechanisms underlying increased drug sensitivity in the high–risk group, we identified pathways specifically enriched in this subgroup by gene set variation analysis (GSVA). A total of fourty–one KEGG pathways were significantly enriched in high–risk patients (FDR < 0.05), including MAPK, TGF–β, and mTOR signaling, ECM–receptor interaction, focal adhesion, regulation of autophagy, and multiple cancer–related pathways (Supplementary Table S3). Notably, several pathways were closely linked to the predicted sensitivities to Bortezomib, Dabrafenib, Talazoparib, and Teniposide. Specifically, since Bortezomib modulates NF–κB and induces ER stress and apoptosis, there is extensive interactive regulation between NF–κB and TGF–β signaling pathway [57]. Dabrafenib targets *BRAF*, a key component of the MAPK signaling pathway, which was markedly activated in the high–risk group [58,59]. Talazoparib, a poly (ADP–ribose) polymerase (*PARP*) inhibitor, exploits synthetic lethality in tumors with impaired DNA repair [60]. The enrichment of autophagy regulation and cell–cell junction pathways (e.g., tight junction, adherens junction) suggested possible epithelial stress and genomic instability, which may sensitize cells to *PARP* inhibition. Teniposide, a topoisomerase II inhibitor, induces DNA double–strand breaks [61]. Its efficacy may be enhanced in tumors with active MAPK, mTOR, or ECM–related signaling, all enriched in the high–risk group. The correlation analysis results indicated that higher MIPM risk scores could increase the sensitivity of CRC cell lines to Dactolisib, Trametinib, Ulixertinib, and so on, but led to increased resistance to several compounds, such as Veliparib, Motesanib, and Erlotinib (*p* < 0.05), which may provide some assistance in the treatment of CRC patients (Figure 8B). In summary, this part might identify promising therapeutic candidates for CRC treatment.

### 3.8. Immune Characteristics Between the High– and Low–Risk Groups

The tumor microenvironment (TME) plays a fundamental role in tumor progression and metastasis [62,63]. First, we found that high–risk patients showed higher immunocyte infiltration degrees in M0 macrophages, M2 macrophages, activated mast cells, and neutrophils; in comparison, the low–risk group showed higher infiltration degrees in activated memory CD4 T cells, regulatory T cells, CD8 T cells, and resting NK cells, etc., illustrating that MIPM might partly reflect TME status (Figure 8C,D).

Subsequently, we evaluated the immune checkpoint inhibitors (ICIs) therapy response of different MIPM subgroups using the tumor immune dysfunction and exclusion (TIDE) approach. An elevated TIDE score represented an increased likelihood of immune escape and was associated with poorer immunotherapy efficacy [45]. Compared with the low–risk group, TIDE scores and T cell exclusion scores in the high–risk group were significantly increased, while T cell dysfunction scores and microsatellite instability (MSI) scores were decreased, indicating greater immune escape potential of high–risk patients, and the fact that they were less likely to benefit from ICI therapy (Figure 8E). We also found that patients with lower TIDE scores consistently had a worse prognosis among four patient groups stratified by MIPM risk scores and TIDE scores, patients with high–risk and low–TIDE really tended to have the highest risk for death (Figure 8F,G).

The expression of immune checkpoints has been recognized as a biomarker for the selection of CRC patients for immunotherapy [64,65]. So, we evaluated the relationships between MIPM risk scores and immune–related features and the expression of key immune checkpoints in sequence (Figure 8H). Based on the results of ESTIMATE method, we found that MIPM risk scores were correlated with stromal scores, immune scores, estimate scores, and tumor purity. Meanwhile, we identified high associations between MIPM risk scores and the expression of three important immune checkpoints (*PD–1*, *PD–L1*, and *CTLA–4*). We also investigated the differences in the expression of immune checkpoints between the high– and low–risk groups, and the results showed that the expression of the three immune checkpoints in the high–risk group was significantly higher than that in the low–risk group, suggesting poor prognosis in the high–risk group due in part to the immunosuppressive environment (Figure 8I).

The individual *PD1* and *PD–L1* responses could be quantified using the immunophenoscore (IPS), which consists of four different scores (molecules (MHCs), effector cells (ECs), suppressor cells (SCs), and immune checkpoints (CPs)) [47,66]. The results illustrated that there were significant differences in all four scores between MIPM subgroups (Figure 8J). Among them, in the high–risk group, MHC IPS, EC IPS, and SC IPS were significantly lower, but CP IPS was significantly higher.

Consistent with our results, numerous previous studies have also confirmed the involvement of MIPM genes in regulating the tumor immune microenvironment or therapy resistance. *C5AR1*, a pleiotropic regulator, promoted CRC initiation by fostering a tumor-supportive immune response and might serve as a potential preventive target [48]. *CCR7* was linked to immune dysregulation and tumorigenesis, with studies highlighting its key role in coordinating immune cell trafficking [67,68]. *ICOS* exerted dual roles in tumor immunity by enhancing cytotoxic T cell activity while also promoting Treg–mediated immunosuppression, thus contributing to both anti– and pro–tumor effects [69,70]. *IL2RB*, essential for T cell proliferation and survival, was closely associated with immune checkpoint signaling in CRC. *IL2RB*–positive immune cells were often linked to immune suppression and T cell exhaustion [51,71]. *NRP1*, highly expressed on Tregs, supported their suppressive function and contributed to immune evasion and therapy resistance by sustaining immunosuppressive signaling and dampening effective T cell responses [72,73]. These results indirectly suggested that MIPM might play a crucial role in predicting immunotherapy outcomes.

## 4. Discussion

Colorectal cancer (CRC) exhibits high heterogeneity, leading to substantial variability in patient survival outcomes [74,75]. A major challenge in treating CRC is metastasis, particularly to the liver, which affects approximately 35–55% of CRC patients and complicates effective treatment strategies [76]. Concurrently, the intricate interplay between the immune system and cancer metastasis adds further complexity to CRC progression. Numerous genetic markers, including mutations in *KRAS*, *BRAF*, and *TP53*, have been identified as prognostic indicators. For instance, studies by Lievre A. et al. and Lee et al. had highlighted the prognostic value of these genetic alterations in CRC [77,78]. However, these markers fail to capture the full complexity of CRC, as they overlook the immune landscape and metastatic potential. This limitation underscores the urgent need to integrate prognostic signatures that include both immunological and metastatic factors. To address this limitation, we employed a bioinformatics methodology to construct the metastasis-based immune prognostic model (MIPM) by integrating CRC single–cell RNA sequencing (scRNA–seq) and bulk data. By leveraging both bulk sequencing for extensive phenotypic and survival data, and scRNA–seq to address cellular heterogeneity, our analysis synergized the strengths of these techniques [79,80].

To develop MIPM, we employed various computational approaches in scRNA–seq data, including signal–to–noise statistics for gene expression comparison and supervised learning to distinguish between primary and metastatic cells. This approach led to the identification of a gene expression signature comprising 4000 genes, which could well distinguish primary and metastatic cells. Further refinement, focusing on high–contribution immune–related genes selected by random forest algorithm, combined with the Cox and Lasso regression analysis to screen prognostic genes, resulted in a concise MIPM with six genes. Our validation confirmed the associations between MIPM and various clinical characteristics, revealing significant differences in MIPM risk scores across subgroups with different clinical features. And, to test the robustness of MIPM, our verification across multiple bulk datasets, we found the effectiveness of MIPM in predicting overall survival (OS) and its significant implications for disease–free survival (DFS) outcomes of patients. Notably, compared to other existing immune–related models, MIPM demonstrated a more robust and superior predictive performance in eleven independent validation datasets (Figure 8K). The efficacy of MIPM extended to predicting chemotherapy benefits, as evidenced in validation datasets. Among these, we highlighted findings not previously discussed, and MIPM demonstrated consistent predictive power in the chemotherapy-naïve subgroup, supporting its robustness independent of treatment status (Figure 8L). It showed a strong correlation with drug sensitivity, and some potential candidate drugs for CRC have also been identified through analysis. Multiple immunoassays revealed the powerful ability of MIPM to predict immunotherapy response. This evidence positioned MIPM as a promising prognostic marker, potentially guiding future CRC metastasis, treatment, and prognosis research.

However, our study still has several limitations. First, the key prognostic features identified in MIPM require further experimental validation to clarify their underlying biological mechanisms, including comprehensive functional characterization through both in vitro and in vivo studies. Second, although OncoPredict enables transcriptome–based drug sensitivity prediction, its extrapolation from GDSC cell lines to clinical CRC samples is constrained by biological differences, including the lack of tumor microenvironment and immune context. These limitations may affect the generalizability of our results and highlight the need for in vivo or patient–derived model validation. Furthermore, CIBERSORT and TIDE analyses provide valuable insights into the immune landscape, but they have limitations in CRC. CRC exhibits pronounced inter– and intra–tumoral heterogeneity. CIBERSORT depends on the predefined signatures of twenty–two immune cell types, which may inadequately capture the complex composition or plasticity of the immune microenvironment in CRC, especially within the microsatellite instability–high (MSI–H) or consensus molecular subtype subgroups. Similarly, TIDE simplifies immune evasion into a few gene markers (e.g., *PD–1*, *CTLA–4*), and its model is mainly trained on melanoma and non-small-cell lung cancer (NSCLC) data, potentially limiting its applicability to CRC due to distinct tumor biology and immune contexts. Currently, publicly available CRC cohorts with both transcriptomic data and clinical response to ICIs are very limited. Most ICI–treated cohorts focus on melanoma, lung, or bladder cancer. Only a few small CRC datasets exist, often with small sample sizes or lacking raw expression data. We acknowledge this as a limitation of our study. To address this, we plan to validate our findings using prospective CRC–ICI cohorts or multi-omics integration in the future.

## 5. Conclusions

In summary, through our bioinformatics approach, integrating single–cell RNA sequencing (scRNA–seq) and bulk data, we constructed metastasis–based immune prognostic model (MIPM). Our comprehensive evaluation highlighted MIPM’s association with metastasis and its predictive accuracy for CRC patient survival. Its integration with clinical variables improved risk prediction accuracy, laying a foundation for further investigation toward clinical relevance. In addition, MIPM integrated genes involved in both immune regulation and metastasis, providing new insights into the interplay between immune microenvironment and tumor dissemination in CRC. This could contribute to a deeper understanding of the molecular mechanisms underlying disease progression and therapeutic resistance. MIPM emerges as a promising prognostic tool, offering new avenues for understanding CRC metastasis and enhancing clinical research.

## Figures and Tables

**Table 1:** Summary of datasets used in this study.

	Series	Platform	Cells/Patients	Samples Treated with Chemo
scRNA–seq	GSE178318	GPL24676	113,331/6	3
Bulk	GSE39582	GPL570	556	233
	GSE159216	GPL17586	171	156
	GSE87211	GPL13497	196	196
	GSE29621	GPL17586	65	38
	GSE72968	GPL570	68	68
	GSE72970	GPL570	124	124
	GSE12945	GPL96	62	–
	GSE17536	GPL570	177	–
	GSE17537	GPL570	55	–
	GSE17538	GPL570	232	–
	GSE37892	GPL570	130	–

**Table 2:** Summary of validation cohorts and MIPM performance.

Series	Platform	Sample Size	N (OS Event)	N (DFS Event)	*p* Value	HR	95% CI	AUC
GSE39582	GPL570	556	187	177	<0.001	2.153	1.603–2.891	0.655
GSE159216	GPL17586	171	108	-	<0.001	1.967	1.347–2.873	0.624
GSE87211	GPL13497	196	28	28	<0.001	3.578	1.460–8.770	0.653
GSE29621	GPL17586	65	25	9	<0.001	5.068	1.811–14.182	0.748
GSE72968	GPL570	68	49	-	0.019	1.925	1.032–3.590	0.559
GSE72970	GPL570	124	92	-	0.049	1.510	1.032–2.273	0.572
GSE12945	GPL96	62	12	4	0.002	5.247	0.705–39.062	0.622
GSE17536	GPL570	177	73	36	<0.001	2.774	1.693–4.543	0.684
GSE17537	GPL570	55	20	19	0.023	2.769	1.132–6.770	0.584
GSE17538	GPL570	232	93	55	<0.001	2.439	1.574–3.780	0.656
GSE37892	GPL570	130	37	-	<0.001	2.870	1.433–5.747	0.621

**Table 3:** Univariate and multivariate Cox regression analysis in validation datasets.

	Univariate Analysis			Multivariate Analysis		
Variables	HR	95% CI	*p* Value	HR	95% CI	*p* Value
GSE39582						
Risk (high vs. low)	2.160	1.615–2.888	<0.001 ***	1.873	1.393–2.518	<0.001 ***
Sex (male vs. female)	1.328	0.990–1.780	0.058 .	1.346	0.999–1.814	0.051 .
Age	1.023	1.011–1.036	<0.001 ***	1.023	1.011–1.036	<0.001 ***
Stage (III/IV vs. I/II)	1.818	1.360–2.431	<0.001 ***	1.815	1.353–2.433	<0.001 ***
GSE17538						
Risk (high vs. low)	2.489	1.623–3.817	<0.001 ***	2.225	1.443–3.429	<0.001 ***
Sex (male vs. female)	1.006	0.659–1.535	0.979	1.019	0.658–1.578	0.934
Age	1.012	0.995–1.029	0.171	1.022	1.005–1.039	0.013 *
Stage (III/IV vs. I/II)	3.563	2.139–5.935	<0.001 ***	3.604	2.146–6.054	<0.001 ***
GSE17536						
Risk (high vs. low)	2.790	1.755–4.436	<0.001 ***	2.598	1.629–4.144	<0.001 ***
Sex (male vs. female)	1.105	0.694–1.759	0.674	1.032	0.630–1.689	0.902
Age	1.006	0.988–1.025	0.492	1.016	0.997–1.034	0.096 .
Stage (III/IV vs. I/II)	4.220	2.387–7.459	<0.001 ***	4.217	2.369–7.506	<0.001 ***

‘***’, *p* < 0.001; ‘*’, *p* < 0.05; ‘.’, 0.05 < *p* < 0.1.

## Data Availability

The data used in this study are openly available in GEO (https://www.ncbi.nlm.nih.gov/geo/browse/?view=series, accessed on 13 June 2025), ImmPort (https://www.immport.org/resources, accessed on 13 June 2025), and GDSC (https://www.cancerrxgene.org/, accessed on 13 June 2025) databases.

## Supplementary Materials

The following supporting information can be downloaded at https://www.mdpi.com/article/10.3390/cimb47080652/s1.

## Abbreviations

The following abbreviations are used in this manuscript:

CRC	Colorectal cancer
MIPM	Metastasis–based immune prognostic model
scRNA–seq	Single–cell RNA sequencing
OS	Overall survival
IRGPI	Immune–related gene prognostic index
TIME	Tumor immune microenvironment
ICIs	Immune checkpoint inhibitors
GEO	Gene Expression Omnibus
UMI	Unique molecular identifier
ssGSEA	Single-sample gene set enrichment analysis
HIRG	High–contribution immune-related genes
K-M	Kaplan–Meier
DCA	Decision curve analysis
ROC	Receiver operating characteristic
DFS	Disease–free survival
IC50	Half maximal inhibitory concentration
GDSC	Genomics of Drug Sensitivity in Cancer
TIDE	Tumor immune dysfunction and exclusion
IPS	Immunophenoscore
QC	Quality control
t-SNE	T–distributed stochastic neighbor embedding
AUC	Area under the ROC
CMS	Consensus molecular subtypes
GSVA	Gene set variation analysis
PDOX	Patient-derived organoid–based xenograft
PARP	Poly (ADP–ribose) polymerase
TME	Tumor microenvironment
MSI	Microsatellite instability
MHC	MHC molecules
EC	Effector cells
SC	Suppressor cells
CP	Immune checkpoints
NSCLC	Non-small-cell lung cancer
