# Supplementary material for: Integrating Primary and Metastatic scRNA–Seq and Bulk Data to Develop an Immune–Based Prognosis Signature for Colorectal Cancer
Source: Curr Issues Mol Biol. 2025 Aug 13;47(8):652. doi: 10.3390/cimb47080652 (PMC12384769; doi:10.3390/cimb47080652)
Supplement: Supplementary file 1 [file cimb-47-00652-s001.zip › fractalfract-3791428-Supplementary Table S1.pdf]

Supplementary Table S1. Marker genes of main cell types.

**Cell Types**	**Marker Genes**
Epithelial Cells	EPCAM
T Cells	CD3D, CD3G
B Cells	CD19, CD79A, MS4A1
Plasma Cells	MZB1
Myeloid Cells	CD68, CD163, CD14, LYZ
Endothelial Cells	CLDN5, CDH5
Mast Cells	TPSAB1, TPSB2, MS4A2
NK Cells	KLRF1, KLRD1, FGFBP2, PRF1
CAFs	FAP, COL1A1, COL3A1, DCN, ACTA2
pDCs	LILRA4, IL3RA
