# Supplementary material for: Integrating Primary and Metastatic scRNA–Seq and Bulk Data to Develop an Immune–Based Prognosis Signature for Colorectal Cancer
Source: Curr Issues Mol Biol. 2025 Aug 13;47(8):652. doi: 10.3390/cimb47080652 (PMC12384769; doi:10.3390/cimb47080652)
Supplement: Supplementary file 1 [file cimb-47-00652-s001.zip › fractalfract-3791428-Supplementary Table S2.pdf]

Supplementary Table S2. Model’s genes and coefficients developed by previous studies.

Model	Model genes	Coefs
IRGPI		
	S100Z	1.016
	BDNF	0.625
	PPARGC1A	-0.405
	TRAF5	0.370
	NOXA1	0.494
	DDIT3	0.249
	AGER	-0.507
	VAV2	0.365
	NMB	0.300
	MC1R	0.430
	TRAF2	-0.830
	CNPY3	0.535
	CTNNB1	-0.555
	TRIM58	0.232
	GLP2R	-0.906
	PTH1R	0.404
	CD36	0.289
Xiao et al.		
	NAT2	-0.32727
	UGT2A3	-0.16282
	STC2	0.34818
	ESM1	0.33961
Zheng et al.		
	CXCL1	-0.32029
	CAT	-0.84476
	PGLYRP2	0.67235
	GRP	0.33094
	ACTG1	-1.4025
	RAF1	-1.0255
Li et al.		
	GLP2R	-0.11758
	NR3C2	-0.06016
	ZC3HAV1L	-0.10207
	SCG2	0.09357
	GDF15	-0.00615
	TG	-0.07016
	ULBP2	0.09061
	PTH1R	0.16894
	UCN	0.50362

STC1	0.20628
UCN3	-0.07878
INHBB	0.16237
ORM1	-0.11964
IL1A	-0.06180
HAMP	0.01244
PCSK1	-0.02415

The risk score calculation formulas for the models are all as follows:

$$\text{RiskScore} = \sum_{i=1}^{n}(\text{Gene Expression}(i) \times \text{Coef}(i))$$
