# Supplementary material for: Integrating Primary and Metastatic scRNA–Seq and Bulk Data to Develop an Immune–Based Prognosis Signature for Colorectal Cancer
Source: Curr Issues Mol Biol. 2025 Aug 13;47(8):652. doi: 10.3390/cimb47080652 (PMC12384769; doi:10.3390/cimb47080652)
Supplement: Supplementary file 1 [file cimb-47-00652-s001.zip › fractalfract-3791428-Supplementary Table S3.pdf]

Supplementary Table S3. Pathways enriched in the high-risk group.

Pathways	FDR
ECM_RECEPTOR_INTERACTION	<0.0001
FOCAL_ADHESION	<0.0001
ADHERENS_JUNCTION	<0.0001
GLYCOSAMINOGLYCAN_BIOSYNTHESIS_CHONDROIT	<0.0001
TGF BETA SIGNALING PATHWAY	<0.0001
ARRHYTHMOGENIC_RIGHT_VENTRICULAR_CARDIO	<0.0001
MELANOMA	<0.0001
HYPERTROPHIC_CARDIOMYOPATHY_HCM	<0.0001
PROSTATE_CANCER	<0.0001
REGULATION_OF_ACTIN_CYTOSKELETON	<0.0001
MTOR_SIGNALING_PATHWAY	<0.0001
DILATED_CARDIOMYOPATHY	<0.0001
PATHWAYS_IN_CANCER	<0.0001
TIGHT_JUNCTION	<0.0001
GAP_JUNCTION	<0.0001
VASCULAR_SMOOTH_MUSCLE_CONTRACTION	<0.0001
GLYCOSAMINOGLYCAN_BIOSYNTHESIS_HEPARAN_S	<0.0001
RENAL_CELL_CARCINOMA	<0.0001
SMALL_CELL_LUNG_CANCER	<0.0001
AXON_GUIDANCE	<0.0001
PANCREATIC_CANCER	<0.0001
PATHOGENIC_ESCHERICHIA_COLI_INFECTION	<0.0001
CIRCADIAN_RHYTHM_MAMMAL	<0.0001
MELANOGENESIS	0.0002
GLIOMA	0.0003
COLORECTAL_CANCER	0.0003
PHENYLALANINE_METABOLISM	0.0013
BLADDER_CANCER	0.0021
DORSO_VENTRAL_AXIS_FORMATION	0.0024
ENDOMETRIAL_CANCER	0.0042
CHRONIC_MYELOID_LEUKEMIA	0.0046
BETA_ALANINE_METABOLISM	0.0054
LEUKOCYTE_TRANSENDOTHELIAL_MIGRATION	0.0055
BASAL_CELL_CARCINOMA	0.0060
HEDGEHOG_SIGNALING_PATHWAY	0.0070
CARDIAC_MUSCLE_CONTRACTION	0.0111
ACUTE_MYELOID_LEUKEMIA	0.0185
REGULATION_OF_AUTOPHAGY	0.0307
GLYCOSAMINOGLYCAN_DEGRADATION	0.0320
FC_GAMMA_R_MEDIATED_PHAGOCYTOSIS	0.0386
MAPK_SIGNALING_PATHWAY	0.0487
